# Research on the Impact and Mechanism for the Inhibition of Micrococcus Catalase Activity by Typical Tetracyclines

**DOI:** 10.1155/2020/5085369

**Published:** 2020-10-13

**Authors:** Luyao Ren, Qian Wang, Yonggang Du, Pengju Xu, Wansong Zong

**Affiliations:** College of Geography and Environment, Shandong Normal University, 1# Daxue Road, Jinan, Shandong 250014, China

## Abstract

As potential inhibitors target to biological enzymes, antibiotics may have certain impacts on the biochemical treatment process. With micrococcus catalase (CAT) served as the target molecule, the impact and inhibition mechanism for typical tetracyclines (TCs) were evaluated. Toxicity experiments showed that TCs had significant inhibition on CAT in the sequence of tetracycline>chlortetracycline>oxytetracycline>doxycycline. To clarify the inhibition mechanism between TCs and CAT which was explored with the assistance of fluorescence spectroscopy and MOE molecule simulation. According to fluorescence analysis, TCs quenched the fluorescence signal of CAT by the mode of static quenching. Combined with toxicity data, it could be presumed that TCs combined with the catalytic active center and thus inhibited CAT. Above presumption was further verified by the molecular simulation data. When TCs combined with the catalytic center of CAT, the compounds have increased combination areas and prominent energy change (compared with the compounds formed by TCs and noncatalytic center recommend by MOE software). IBM SPSS statistics showed that TC toxicity positively correlated with the hydrogen bonds such as O^13^→Glu_252_, O^1^←Arg_195_, and O^6^→Asp_249_, but negatively correlated with the hydrogen bonds such as O^10^→Pro_363_, O^10^→Lys_455_, and O^12^ → Asn_127_. TC toxicity also positively correlated with the ion bonds ofN^4^-Glu_252_, but negatively correlated with the ion bonds of N^4^-Asp_379_. Hydrogen bonds and ion bonds for above key sites were closely related to the inhibition effect of TCs on CAT.

## 1. Introduction

TCs have been widely used in animal husbandry and aquaculture as the advantages of low-cost and broad antibacterial spectrum [1–4]. Because of their low bioavailability [5], 50%-80% of TCs enter the environment as contaminants without being metabolized [6]. Residual antibiotics have posed serious threat to environment [7, 8]. In addition, the abuse of antibiotics leads to the emergence of numerous drug-resistant pathogens [9]. Drug residues and related resistance genes have attracted the attention of international environmental scholars [10, 11].

In view of TC biotoxicity, controls on their levels become of great importance. At present, the main treatment methods are the chemical method and biochemical method [12–14]. Compared with the chemical method, the biochemical method has the advantages of low-cost and easy engineering, thus has become the mainstream technology [15]. However, the biochemical method has the problems of difficult microbial domestication and not impact resistance [16, 17]. As potential inhibitors of biological enzymes, antibiotics have certain impacts on the biochemical treatment process. Accordingly, evaluating the inhibition effect of antibiotics on biological enzymes is necessary [17, 18].

Biological enzymes include hydrolases, oxidoreductases, and transferases, which are mainly derived from bacteria, fungi, protozoa, and algae. Catalase (CAT) is an oxidoreductase with iron porphyrin as a prosthetic group [19, 20]. It promotes the decomposition of H_2_O_2_ into molecular oxygen and water, removes hydrogen peroxide from the body, and protects cells from H_2_O_2_ toxicity [20, 21]. It is one of the key enzymes in the biological defense system and plays an important role in the biochemical treatment of TC wastewater [22]. And the CAT activity directly reflects the biological activity of the treatment system and affects the whole sewage treatment process once inhibited [16, 17]. Therefore, clarifying the impact and inhibition mechanism of TCs on the CAT activity not only helps to understand the discrepant toxicity of TCs but also has practical guiding significance for engineering measures to solve TC pollution.

To clarify the inhibition mechanism, micrococcus catalase was selected to evaluate the impact and inhibition mechanism by typical TCs. The interaction between TCs and CAT was explored with the assistance of fluorescence spectroscopy and molecular docking simulation. According to fluorescence spectroscopy, the impact of TCs on CAT characteristic fluorescence spectra could be obtained to confirm their quenching mechanism. In addition, the inhibition mechanism could be explained by the docking data: combination area, energy change, hydrogen bonds, and ion bonds for key sites. This study offers a comprehensive cognition on TC toxicity regulation and provides valid theoretical support to control their potential risk.

## 2. Materials and Methods

### 2.1. Materials

Tetracycline, chlortetracycline, oxytetracycline, and doxycycline were purchased from Meilun Biotechnology Co., Ltd. (Dalian, China). Micrococcus CAT was purchased from Sigma (Saint-Quentin Fallavier, France). Hydrochloric acid, sulfuric acid, sodium hydroxide, H_2_O_2_, sodium dihydrogen phosphate, and disodium phosphate were purchased from Sinopharm (Shanghai, China). Methanol and ethanol were obtained from Fisher Chemicals (Fair Lawn, NJ, USA).

### 2.2. Toxicity Test for the Interaction between TCs and CAT

Referring to Yang's ultraviolet spectrophotometry [23], the biological toxicity of TC target to CAT was evaluated. 2 mL of 0.2 mol/L phosphate buffer (pH 7.8), 1 ml of 400 mg/L CAT solution, different amounts (100 mg/L-1500 mg/L) of TC solution, and 5 mL of 0.3% H_2_O_2_ were sequentially added to 50 mL centrifuge tubes. Tetracycline was solubilized with ethanol, chlortetracycline was solubilized with methanol, oxytetracycline was solubilized with 0.2 mol/L hydrochloric acid solution, and doxycycline was solubilized with 0.1 mol/L sodium hydroxide solution. After shaking for 20minutes, sulfuric acid was added immediately and then measured at 240 nm by UV spectrophotometer. The toxicity of TCs on CAT was reflected by measuring the absorbance of the remaining H_2_O_2_. The toxicity of TCs was calculated by the formula of A_sample_-A_control_. A_control_ and A_sample_ were the absorbance of the reference sample (without TCs) and test sample at 240 nm, respectively.

### 2.3. Fluorescence Spectroscopy for the Interaction between TCs and CAT

Fluorescence spectra were measured by a three-dimensional fluorescence spectrometer (Hiachi Limited, F-7000). Firstly, 2 mL of 0.2 mol/L phosphate buffer (pH 7.8), 1 mL of 400 mg/L CAT solution, and 1 ml different amounts (0 mg/L-1500 mg/L) of TC solution were sequentially added to 50 mL colorimetric tubes. In addition, 2 mL of 0.2 mol/L phosphate buffer (pH 7.8) and 1 mL of 1500 mg/L TC solution were added to another 50 mL colorimetric tube, then make the final volume to 50 ml with ultra-pure water, and mixed and kept for 30 min. The fluorescence spectra of samples were measured at an excitation wavelength of 276 nm and an emission wavelength range of 287-380 nm. Fluorescence quenching type was calculated by the dynamic quenching formula of Stern-Volmer: *F*_0_/*F* = 1 + *K*_sv_[*Q*] = 1 + *K*_*q*_*τ*_0_[*Q*] [24]. *F*_0_ and *F* were the fluorescence intensity in the absence and presence of the quencher. *K*_sv_, [*Q*], *K_q_*, and *τ*_0_ were the quenching constant, the concentration of the quencher, the fluorescence quenching rate constant, and the average lifetime of the fluorescent molecules in the absence of the quencher. For biomacromolecules, *τ*_0_ = 10^−8^ s.

### 2.4. Molecular Simulation for the Interaction between TCs and CAT

Molecular docking simulation was performed with Molecular Operating Environment software (MOE, version 16.09). The main experimental steps are as follows: the model forCAT was obtained from the Protein Data Bank (PDB code 1HBZ, http://www.rcsb.org/pdb/home/home.do) [25]. Then, receptor CAT was minimized for energy optimization. MOE parameters were set as follows: amber10 EHT, solvation r-field, temperature 25.0°C, pH 7.4, and salinity 0.05 M. The interactions between TCs and CAT (combination areas, energy change, hydrogen bonds, and ion bonds for main interaction sites) were simulated by the “molecular hole method” to clarify the molecular mechanism for the discrepant inhibition of TCs on CAT. The detailed operating procedures can be found in supplementary information.

## 3. Results and Discussions

### 3.1. Biological Toxicity Evaluation of TC target to CAT

**Figure 1:**
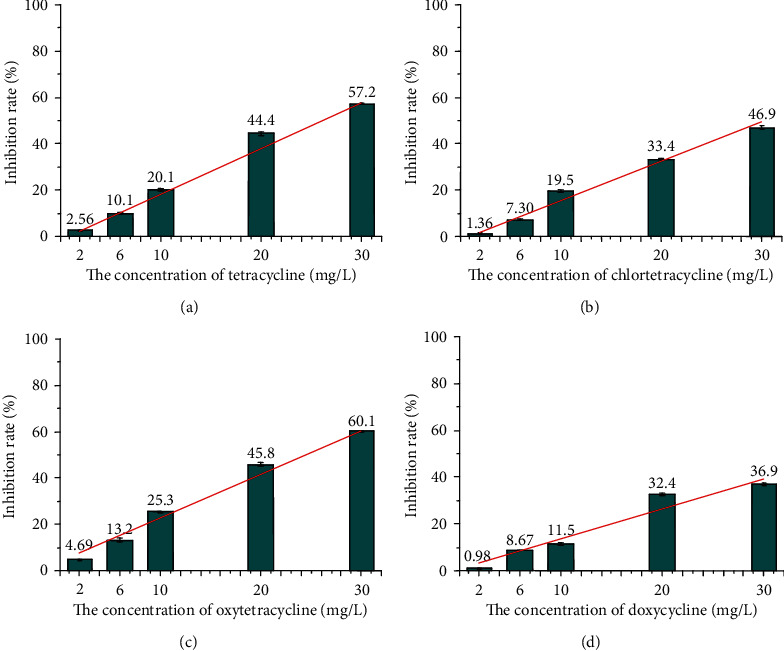
Inhibition effect of typical TC target to the CAT activity: (a) tetracycline, (b) chlortetracycline, (c) oxytetracycline, and (d)doxycycline.

CAT catalyzes the decomposition of H_2_O_2_ into oxygen and water. Therefore, the toxicity of TCs on CAT could be reflected by measuring the absorbance of the remaining H_2_O_2_.  Figure 1 shows that the activity of CAT was inhibited by TCs in varying degrees. With the increase of TC concentration, the inhibition effect on CAT was on the rise, showing a strong dose-effect relationship. Based on the slope of linear fitting between inhibition rate and TC concentration, the toxicity of TCs on CAT could be obtained in the sequence of tetracycline>oxytetracycline>chlortetracycline>doxycycline. Accordingly, specific inhibition mechanisms and interspecies discrepancies about the interaction between TCs and CAT deserved further attention.

### 3.2. The Fluorescence Intensity Effect of TC Target to CAT

**Figure 2:**
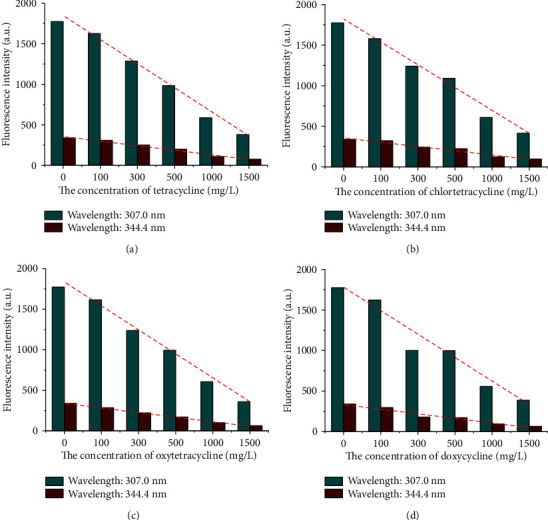
Inhibition effect of different TCs on CAT fluorescence intensity: (a) tetracycline, (b) chlortetracycline, (c) oxytetracycline, and (d) doxycycline.

Fluorescence spectroscopy can be to study the interaction between proteins and small molecules. When TCs interact with CAT, the fluorescence intensity of CAT will show a dose-effect relationship with TC concentration [26]. To explore the interaction mechanism, the fluorescence spectrum of CAT (with or without TCs) was obtained. Figure S1 shows the inhibition effect of tetracycline (served as an example) on the fluorescence spectra of CAT. The fluorescence intensity of CAT with TC concentration was plotted to intuitively evaluate (excitation wavelength: 276 nm, emission wavelengths: 307.0 nm and 344.4 nm, respectively, see Figure 2). With the increasing concentration of TCs, the fluorescence intensity of CAT showed a significant decrease trend. In addition, the interaction was assumed to be dynamic quenching and then calculated by the dynamic quenching formula of Stern-Volmer: *F*_0_/*F* = 1 + *K*_sv_[*Q*] = 1 + *K*_*q*_*τ*_0_[*Q*] [24]. The collision quenching constant due to the maximum diffusion control of biomacromolecules by various quenchers was 2.0 × 10^10^ Lmol^−1^ s^−1^. Since quenching constants in Table 1 were much larger than above *K*_*q*_, the fluorescence quenching of TCs on CAT was caused by static quenching with complexes formed. Combined with toxicity data, the activity of CAT was inhibited to different degrees by TCs, so it was preliminarily judged that TCs and CAT were static quenching in catalytic active center.

### 3.3. The Discrepant Molecular Mechanism for the Interaction between TCs and CAT

#### 3.3.1. The Interaction Models for TC-CAT Complexes

Based on the toxicity experiment and fluorescence spectroscopy experiment, the interaction for TCs to CAT could be preliminary explained, but the specific molecular mechanism was not clarified. According to molecular docking simulation, the interaction models between TCs and CAT could be obtained, and the discrepant inhibition mechanism could be clarified.

**Figure 3:**
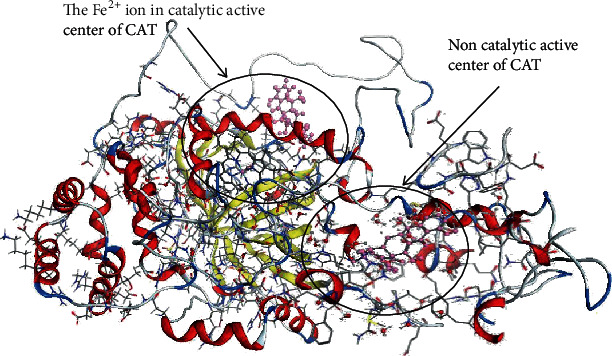
The stereoscopic interaction structure model for TC-CAT complexes (with tetracycline-CAT complexes served as the example).

CAT is a binding enzyme with iron porphyrin as a prosthetic group in the catalytic active center. The stereoscopic structure model for CAT was obtained from the Protein Data Bank (PDB code 1HBZ, catalase from micrococcus lysodeikticu http://www.rcsb.org/pdb/home/home.do) [25]. Based on the *molecular hole method* of MOE software, the interaction models between TCs and CAT in the iron porphyrin catalytic active center and no-catalytic active center were obtained (see Figure 3, with tetracycline-CAT served as the example). Noncatalytic active center was the largest likelihood combination center recommended by software (Compute-Site Finder...-Apply-Select the largest likelihood combination center-Dummies-Yes-Close). Besides, the ligand interaction diagram could provide the specific interaction sites between TCs and CAT (see Figure S2, the order of each atomic name was automatically sorted by software). Accordingly, combination areas and energy changes for different TC-CAT complexes, the hydrogen bonds, and ion bonds for key sites could also be obtained. By comparing on the correlation for changed parameters with toxicity, the discrepant molecule mechanism of TCs on CAT could be clarified.

### 3.4. Combination Areas and Energy Changes For different TC-CAT Complexes

The interaction between TCs and CAT usually involved in changed combination area and energy. To further clarify the combination site, relevant parameters were obtained by MOE, and the rate of combination areas to small molecule areas and energy changes for TC-CAT complexes at different combinatiolizan sites were calculated (see Table 2). Combination area ratio and energy change in the catalytic active center of CAT were higher than those in the noncatalytic active center. Therefore, TCs interacted with CAT the in catalytic active center.

**Figure 4:**
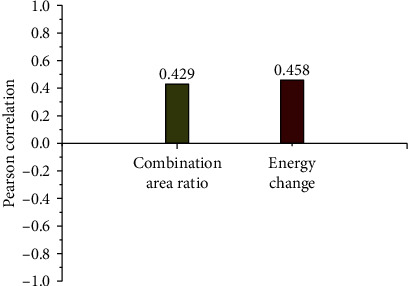
The correlation between TC toxicity and combination area ratio/energy change in the catalytic active center.

Based on data in the catalytic activity center, the combination area ratio for different TC-CAT complexes was determined as follows: chlortetracycline>tetracycline=oxytetracycline>doxycycline. Besides, the energy change in the catalytic active center for different TCs combined with CAT had different decreased in the sequence of chlortetracycline>oxytetracycline>tetracycline>doxycycline. Combined with toxicity data, combination area ratio and energy change were not well correlated with TC toxicity. The combination area and energy change of chlortetracycline were the largest, which could be related to its special chlorine atom structure. Furthermore, the correlation between TC toxicity and combination area ratio/energy change was evaluated by IBM SPSS statistics (see Figure 4). The results showed that the combination area ratio and energy change were basically positively correlated with TC toxicity. Accordingly, it could be served as the reference indexes to evaluate the interaction of TCs on CAT.

### 3.5. The Hydrogen Bonds for Different TC-CAT Complexes

**Figure 5:**
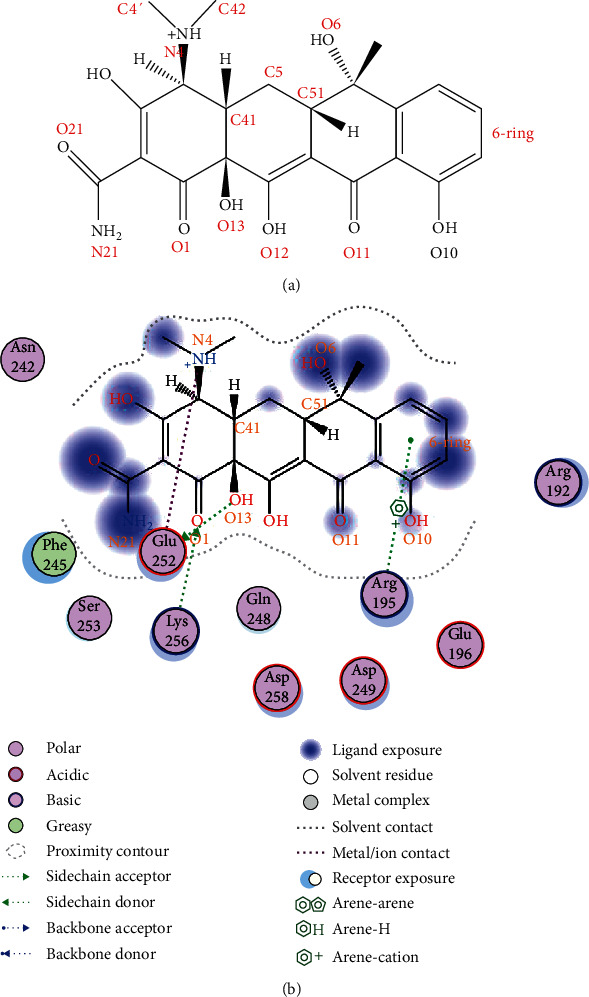
The general structure and the ligand interaction diagram: (a)the general structure of TCs; (b) The ligand interaction diagram between tetracycline and CAT (with one of them as an example, the order of each atomic name was automatically sorted by software).

The specific interaction sites between TCs and CAT can be seen through the ligand interaction diagram provided by the MOE software (see Figure 5). Accordingly, combination areas and energy changes for different TC-CAT complexes, the hydrogen bonds, and ion bonds for key sites could also be obtained. By comparing on the correlation for changed parameters with toxicity, the discrepant molecule mechanism of TCs on CAT could be clarified.

**Figure 6:**
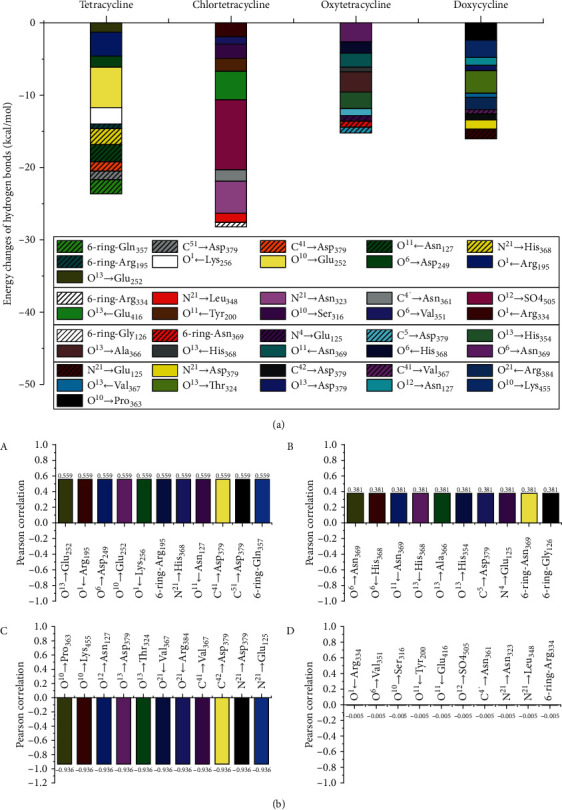
Molecular simulation information of the hydrogen bonds for TC-CAT complexes: (a) energy changes of the hydrogen bonds for TC-CAT complexes. (b) The correlation between TC toxicity and hydrogen bonds for main interaction sites: (a) 0.5 < positive correlation < 1, (b) 0 < positive correlation < 0.5, (c) −1 < negative correlation < −0.5, and (d) −0.5 < negative correlation < 0.

Simulation information of the hydrogen bonds for TC-CAT complexes was shown in Figure 6(a).The total hydrogen bonds for different TC-CAT complexes were determined as follows: chlortetracycline>tetracycline>doxycycline>oxytetracycline. Combined with toxicity data, the downward trend for total hydrogen bonds was not well correlated with the decreased toxicity of TCs. As TCs had multiple potential hydrogen bonding sites with CAT, the hydrogen bonds for single interaction sites were also obtained. Different TCs interacted with CAT to form discrepant hydrogen bonds with different strength. Accordingly, the hydrogen bonds were also important factors to cause the discrepant inhibition effect of TCs on CAT.

**Figure 7:**
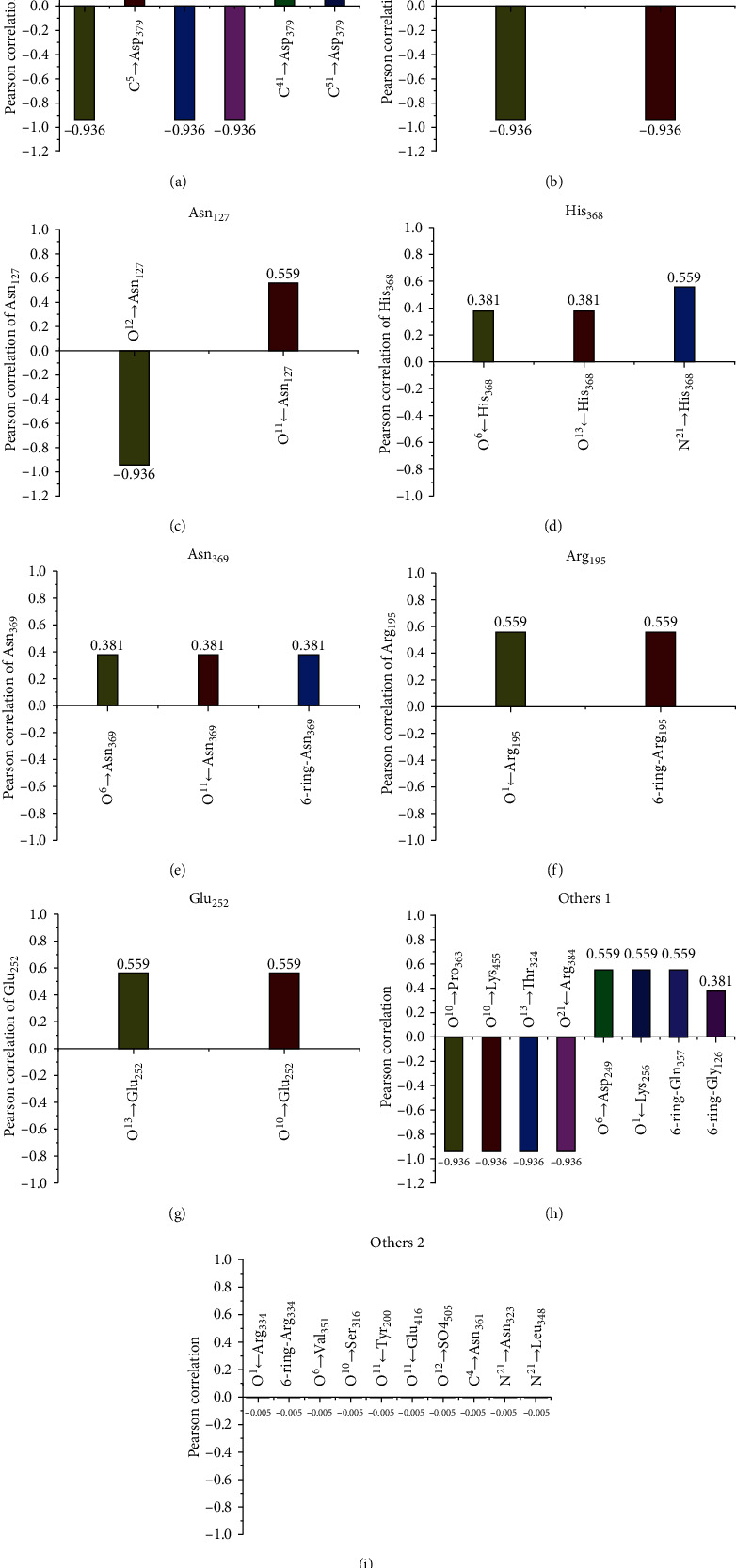
Pearson correlation between TC toxicity and hydrogen bonds for main interaction sites: (a) Asp_379_, (b) Val_367_, (c) Asn_127_, (d) His_368_, (e) Glu125, (f)Asn_369_, (g) Arg_195_, (h) Glu_252_, and (i) other correlated amino acid residues.

To clarify the discrepant inhibition effect, the correlation between TC toxicity and hydrogen bonds for main interaction sites was further evaluated by IBM SPSS statistics.  Figure 6(b) shows that TC toxicity was positively correlated with the hydrogen bonds of O^13^→Glu_252_, O^1^←Arg_195_, O^6^→Asp_249_, O^10^→Glu_252_, O^1^←Lys_256_, 6-ring-Arg_195_, N^21^→His_368_, O^11^←Asn_127_, C^41^→Asp_379_, C^51^→Asp_379_, 6-ring-Gln_357_, O^6^→Asn_369_, O6←His_368_, O^11^←Asn_369_, O^13^←His_368_, C^5^→Asp_379_, N^4^→Glu_125_, O^13^→Ala_366_, O^13^→His_354_, 6-ring-Asn_369_, and 6-ring-Gly_126_, but negatively correlated with O^10^→Pro_363_, O^10^→Lys_455_, O^12^→Asn_127_, O^13^→Asp_379_, O^13^→Thr_324_, O^13^←Val_367_, O^21^←Arg_384_, C^41^→Val_367_, C^42^→Asp_379_, N^21^→Asp_379_, and N^21^→Glu_125_. By statistical analysis, Figure 7(a) shows that the interaction between TCs and CAT was closely related to Asp_379_ residues in CAT, including six hydrogen bonds of C^41^→Asp_379_, C^51^→Asp_379_, O^13^→Asp_379_, C^5^→Asp_379_, C^42^→Asp_379_, abnd N^21^ → Asp_379_. Besides, the correlation sequence of residues including 2-3 interaction sites was verified as follows (Figures 7(b)–7(g)): Val_367_ (O^13^←Val_367_, C^4A^→Val_367_)>Asn_127_ (O^11^←Asn_127_, O^12^→Asn_127_)>His_368_ (O^6^←His_368_, N^21^→His_368_, O^13^←His_368_)>Glu_125_ (N^4^→Glu_125_, N^21^→Glu_125_)>Asn_369_ (O^6^→Asn_369_, O^11^←Asn_369_, 6-ring-Asn_369_)>Arg195 (O^1^←Arg_195_, 6-ring-Arg_195_)>Glu_252_ (O^1C^→Glu_252_, O^10^→Glu_252_).  Figure 7(h) shows that other correlated amino acid residues including only one interaction sites (O^6^→Asp_249_, O^1^←Lys_256_, 6-ring-Gln_357_, O^10^→Pro_363_, O^10^→Lys_455_, O^13^→Thr_324_, O^21^←Arg_384_) were also important parameters to evaluate inhibition mechanism of TCs on CAT. However, Gly_126_, Arg_334_, Val_351_, Ser_316_, Tyr_200_, Glu_416_, So4_505_, Asn_361_, Asn_323_, and Leu_348_ residues were less correlated with TC toxicity (see Figure 7(i)). Combined with the simulation information and related IBM SPSS statistics, changes for above hydrogen bonds could serve as the key indexes to evaluate the interaction of TCs on CAT.

**Figure 8:**
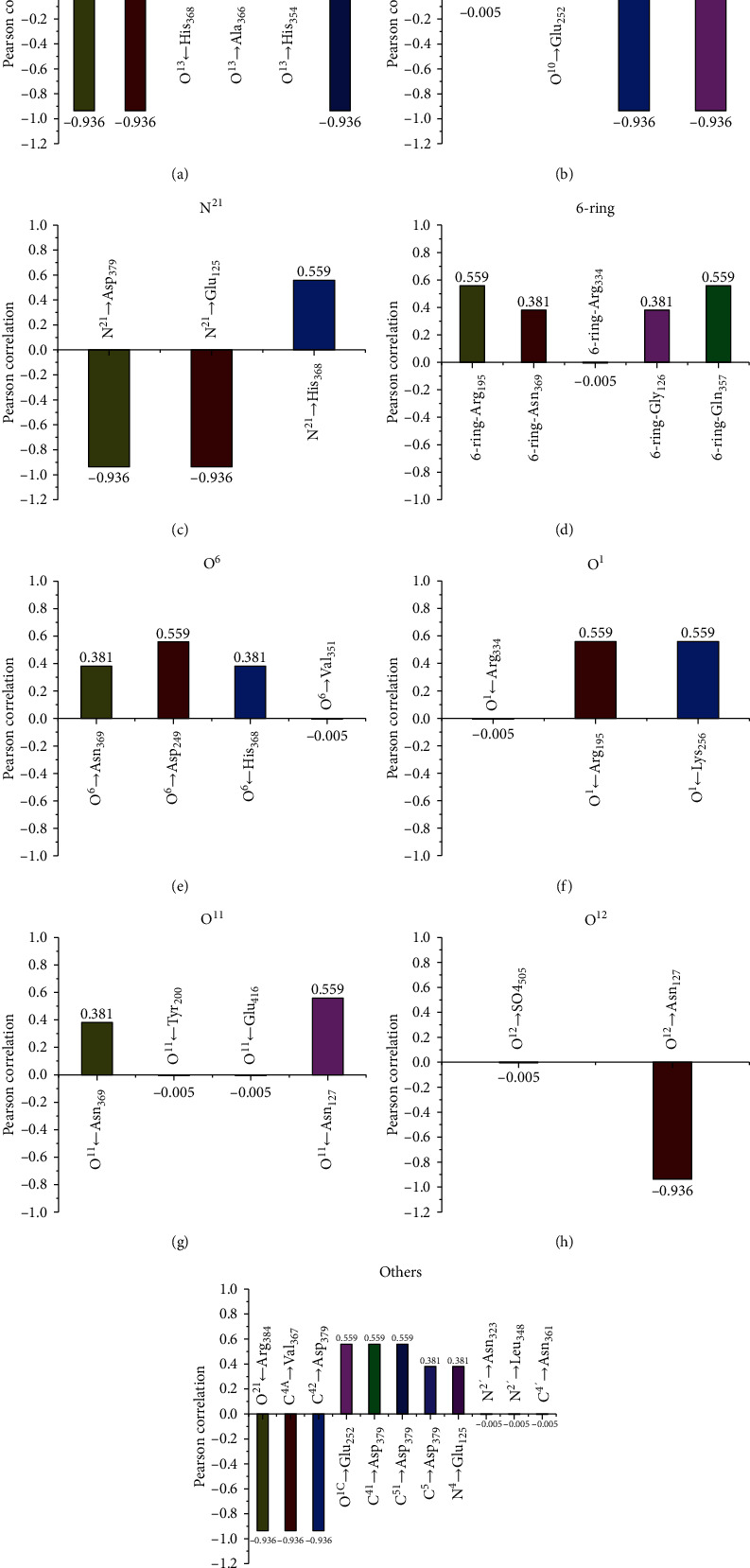
Pearson correlation between TC toxicity and hydrogen bonds for TC main interaction sites: (a) O^13^, (b) O^10^, (c) N^21^, (d) 6-ring, (e) O^6^, (f) O^1^, (g) O^11^, (h) O^12^, and (i) others.

According to statistical analysis, hydrogen bonds related to the key sites of TCs also were ranked (see Figure 8). The interaction between TCs and CAT was closely related to O^13^, involving in hydrogen bonds O^13^→Asp_379_, O^13^→Thr_324_, O^13^←His_368_, O^13^→Ala_366_, O^13^→His_354_, and O^13^←Val_367_. In addition, the correlation sequence of key sites was verified as follows (Figures 8(b)–8(g)): O^10^ (O^10^→Ser_316_, O^10^→Glu_252_, O^10^→Pro_363_, O^10^→Lys_455_)>N^21^ (N^21^→Asp_379_, N^21^→Glu_125_, N^21^→His_368_)>6-ring (6-ring-Arg_195_, 6-ring-Asn_369_, 6-ring-Arg_334_, 6-ring-Gly_126_, 6-ring-Gln_357_)>O^6^ (O^6^→Asn_369_, O^6^→Asp_249_, O^6^←His_368_, O^6^→Val_351_)>O^1^ (O^1^←Arg_334_, O^1^←Arg_195_, O^1^←Lys_256_)>O^11^ (O^11^←Asn_369_, O^11^←Tyr_200_, O^11^←Glu_416_, O^11^←Asn_127_)>O^12^ (O^12^→So4_505_, O^12^→Asn_127_).  Figure 8(i) shows the hydrogen bonds related to only one interaction site of CATs (O^21^←Arg_384_, C^4A^→Val_367_, C^42^→Asp_379_, O^1C^→Glu_252_, C^41^→Asp_379_, C^51^→Asp_379_, C^5^→Asp_379_, N^4^→Glu_125_), which also cannot be ignored. Combining MOE simulation information and related IBM SPSS statistics, these closely related hydrogen bond changes can be used as a key indicator to evaluate the interaction between TCs and CAT and are also very important for the regulation of TC toxicity.

### 3.6. The Ion Bonds for Different TC-CAT Complexes

**Figure 9:**
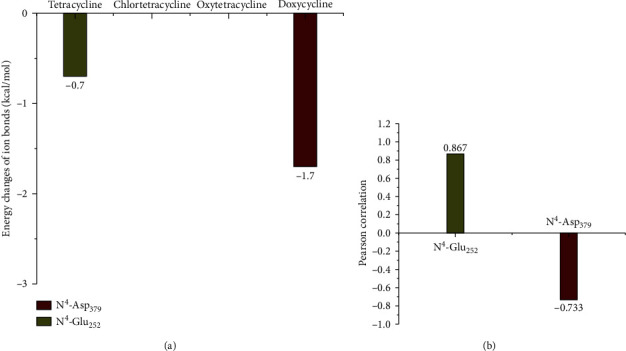
Molecular simulation information of the ion bonds for TC-CAT complexes: (a) energy changes of the ion bonds for TC-CAT complexes; (b) the correlation between TC toxicity and ion bonds.

Simulation information for the ion bonds of TC-CAT complexes were shown in Figure 9(a). Combined with toxicity data, the energy changes of total ion bonds for different TC-CAT complexes had no correlation with toxicity. In addition, the ion bonds were all formed by N_4_ in TCs and amino acids in CAT; therefore, the N^4^ of the two TCs was important to change the activity of CAT. Furthermore, the correlation between TC toxicity and ion bonds for main interaction sites was evaluated by IBM SPSS statistics.  Figure 9(b) shows that the toxicity of TCs was positively correlated with the interaction for N^4^ with Glu_252_ residues, but negatively correlated with the interaction for N^4^ with Asp_379_ residues. In conclusion, above interactions were not only the key to regulate the toxicity of TCs but also important to keep the activity of CAT.

## 4. Conclusions

To clarify the toxicity inhibition mechanism for the discrepant interaction of TCs on CAT, the toxicity of four typical TCs (tetracycline, chlortetracycline, doxycycline, oxytetracycline) on CAT was evaluated. The inhibition sequence was verified as follows: tetracycline>chlortetracycline>oxytetracycline>doxycycline. With the assistance of fluorescence spectroscopy and MOE molecule simulation, the interactions between TCs and CAT were further evaluated. Fluorescence spectroscopy showed that the reduced fluorescence intensity of CAT should be attributed to static quenching between TCs and CAT in the catalytic active center. Molecular simulation showed that combination sites for TC-CAT complexes were in the catalytic active center by calculating the combination area ratio and energy change in the catalytic active center/noncatalytic active center. Combined with toxicity data, the combination area ratio and energy change in the catalytic active center had basically positive correlation with TC toxicity. In addition, combined with the simulation information and related IBM SPSS statistics, TC toxicity was positively correlated with the hydrogen bonds of O^13^→Glu_252_, O^1^←Arg_195_, and O^6^→Asp_249_, but negatively correlated with O^10^→Pro_363_, O^10^→Lys_455_, and O^12^→Asn_127_. By statistical analysis, the interaction between TCs and CAT was closely related to Asp_379_, Glu_252_, Arg_195_, Asn_127_, Val_367_, His_368_, Asn_369_, Asp_249_, Lys_256_, Gln_357_, Pro_363_, Lys_455_, Thr_324_, and Arg_384_ residues in CAT. Besides, TC toxicity was positively correlated with the ion bonds of N^4^-Glu_252_, but negatively correlated with the ion bonds of N^4^-Asp_379_. In conclusion, above interactions could be used as important indexes to evaluate the inhibition mechanism of TCs on the CAT activity. The regulation of TC toxicity could be achieved by weakening or strengthening the hydrogen bonds and ion bonds for some key interaction sites.

## Figures and Tables

**Table 1:** Stern-Volmer quenching constants for the quenching of TCs on CAT at emission wavelengths of 307.0 nm and 344.4 nm, respectively.

TCs		*K*_*q*_ (10^12^ Lmol^−1^ s^−1^, 307.0 nm)	*K*_*q*_ (10^12^ Lmol^−1^ s^−1^, 344.4 nm)
Tetracycline	100 mg/L	2.04	2.19
	300 mg/L	2.81	2.59
	500 mg/L	3.58	3.15
	1000 mg/L	4.49	4.67
	1500 mg/L	5.45	5.05
Chlortetracycline	100 mg/L	2.96	1.25
	300 mg/L	3.43	3.22
	500 mg/L	3.00	2.51
	1000 mg/L	4.58	4.23
	1500 mg/L	5.26	4.08
Oxytetracycline	100 mg/L	2.27	4.41
	300 mg/L	3.33	4.01
	500 mg/L	3.62	4.52
	1000 mg/L	4.44	5.46
	1500 mg/L	6.05	6.63
Doxycycline	100 mg/L	2.07	3.16
	300 mg/L	5.72	6.57
	500 mg/L	3.45	4.32
	1000 mg/L	4.85	5.81
	1500 mg/L	5.30	6.18

**Table 2:** Combination area ratio and energy change for TC-CAT complexes at different combination sites.

TCs	Combination area ratio for catalytic active center	Combination area ratio for noncatalytic active center	Energy change for catalytic active center (kcal/mol)	Energy change for noncatalytic active center (kcal/mol)	Combination sites
Tetracycline	58%	53%	|-5.9784|	|-3.2641|	Catalytic active center
Chlortetracycline	62%	57%	|-7.9117|	|-4.8341|	
Oxytetracycline	58%	56%	|-6.9473|	|-4.5725|	
Doxycycline	55%	53%	|-3.5510|	|-3.3425|	

## Data Availability

The datasets, codes, and corresponding results are available at https://figshare.com/articles/Catalase_correlation_zip/12398684.
