# Supplementary material for: Research on the Impact and Mechanism for the Inhibition of Micrococcus Catalase Activity by Typical Tetracyclines
Source: Biomed Res Int. 2020 Oct 13;2020:5085369. doi: 10.1155/2020/5085369 (PMC7603550; doi:10.1155/2020/5085369)
Supplement: Supplementary Materials — Supplementary information. [file 5085369.f1.docx]

**Supplementary information**

**Experimental steps for molecular docking simulation**

The main experimental steps of molecular docking simulation are as follows:

Functional enzyme used in molecular docking studies is taken from Protein Data Bank（http://www.rcsb.org/pdb/home/home.do），the PDB code are 1HBZ. Use MOE software to dock 4 kinds of TCs with CAT.

**Molecular hole technology**：

1. Open the software and import CAT.
2. Set the parameters, Amber's force field is 10: EHT; Solvation: R-Field; 25.0℃; pH:7.4; salinity:0.05 M, perform overall energy output in “Compute/Docking...”.
3. Energy optimization. Click “QuickPrep/Minimized”， achieve optimal energy for binding between ligand and receptor.
4. Select the bonding area. Compute-Site Finder...-Apply-Select the appropriate docking area (generally select the first 10 chains, the non-catalytic center is the largest possible bonding center recommended by the software)-Dummies-Yes-Close.
5. Import small molecules: TCs. Minimized, File-Open-select the small molecule.
6. Docking. “Compute-Dock...”. Four conjugates of TCs and functional enzymes were obtained (the software automatically screened out the five optimal docking results, and the final binding area of TCs and functional enzymes can be determined by comparing and analyzing the relevant parameters of each conjugate). Import the five results into the software.
7. “Compute/Docking...”: the module then makes the overall energy output.
8. “Compute/Ligand Interactions...” Output action energy, action hydrogen bond, ion bond and other data.
9. Combine the output of the area：In the interface of MOE, create a new Database, enter conjugate, CAT, antibiotic molecule in sequence, calculate in the DBV interface. The sum of the area of the CAT and the antibiotic molecule minus the area of the conjugate and then divided by 2 is the binding area of the ligand and the receptor dock.

**Figure S1**

Figure S1: Inhibition effect of TCs on CAT fluorescence intensity (with tetracycline served as an example). Conditions: CAT 2 mg/L; tetracycline: a, 0 mg/L; b, 2 mg/L; c, 6 mg/L; d, 10 mg/L e, 20 mg/L; f, 30 mg/L; g: 30 mg/L (without CAT).

**Figure S2**

Figure S2: The ligand interaction diagram between TCs and CAT: (A) chlortetracycline; (B) oxytetracycline; (C) doxycycline. (The order of each atomic name was automatically sorted by software).
